# Automated droplet reactor for the synthesis of iron oxide/gold core-shell nanoparticles

**DOI:** 10.1038/s41598-020-58580-9

**Published:** 2020-02-03

**Authors:** Christian D. Ahrberg, Ji Wook Choi, Bong Geun Chung

**Affiliations:** Department of Mechanical Engineering, Sogang University, Seoul, Korea

**Keywords:** Biological techniques, Biotechnology

## Abstract

Core-shell nanoparticles are promising candidates for theranostic drugs, as they combine different intrinsic properties with a small size and large surface area. However, their controlled synthesis, or the screening and optimization of synthesis conditions are often difficult and labor intensive. Through the precise control over mass and heat transfer, and automatization possibilities, microfluidic devices could be a solution to this problem in a lab scale synthesis. Here, we demonstrate a microfluidic, capillary, droplet reactor for the multi-step synthesis of iron oxide/gold core-shell nanoparticles. Through the integration of a transmission measurement at the outlet of the reactor, synthesis results can be monitored in a real-time manner. This allowed for the implementation of an optimization algorithm. Starting from three separate initial guesses, the algorithm converged to the same synthesis conditions in less than 30 minutes for each initial guess. These conditions resulted in diameter for the iron oxide core of 5.8 ± 1.4 nm, a thickness for the gold shell of 3.5 ± 0.6 nm, and a total diameter of the core-shell particles of 13.1 ± 2.5 nm. Finally, applications of the iron oxide/gold core-shell nanoparticles were demonstrated for Surface Enhanced Raman Spectroscopy (SERS), photothermal therapy, and magnetic resonance imaging (MRI).

## Introduction

The small size and large surface area of nanoparticles provides interactions with biological systems that conventional bulk materials cannot provide. Core-shell nanoparticles offer access to a wider range of properties through a combination of two different materials. Through this, a material for the treatment of a disease can be efficiently combined with another material with a monitoring functionality, making these core-shell nanoparticles interesting candidates for theranostic applications^1^. One example of such a material is iron oxide/gold core-shell nanoparticles, which can be used as a theranostic material in cancer therapy. The magnetic core of these particles can be used as a contrast agent for magnetic resonance imaging (MRI)^2–5^, or alternatively for magnetic induced hyperthermia^6,7^. The gold shell increases the biocompatibility of the particles and can be used for photothermal therapy^3^, or modified for drug delivery^5,8–10^. Furthermore, these core-shell nanoparticles can be used for Surface Enhanced Raman Spectroscopy (SERS)^11–13^. Despite the large number of applications, batch synthesis of nanoparticles often suffers from batch-to-batch variations, inhomogeneity in the reaction environment, and requires large labor effort for optimization of reaction conditions^14^. Hence, the alternative platforms for synthesizing core-shell nanoparticles on a lab-scale are required to address these issues.

Microfluidic reactors with their sub-millimeter dimensions offer solutions to these problems. Their small dimensions allow for fast, controllable heat and mass transfer, leading to a homogeneous reaction environment^15,16^. The ability to operate these microfluidic reactors in a continuous, automated manner increases the reproducibility of synthesis results^17^. Compartmentalization of the reaction into droplets within the microfluidic device further provides rapid mixing of reagents and high control over the residence time in the reactor^18^. In addition, segmentation into droplets prevents direct contact of the reagents with the channel walls of the microfluidic device, preventing fouling of the reactor. Due to these advantages, the synthesis of several different nanoparticles in microfluidic devices has been shown using continuous and segmented flow. For example, the silver nanoparticles have been synthesized in microfluidic devices using continuous flow^19^, as well as in droplet flow^20,21^. Furthermore, the silica and gold nanoparticles have been synthesized within droplet flow microfluidic devices, demonstrating that narrower size distributions and higher yields can be achieved compared to batch synthesis^22,23^. Capillary reactors can be used as an alternative to microfluidic devices, since they offer similar advantages regarding mass and thermal transport. However, they can be fabricated from capillary tubing without requiring specialized equipment or a cleanroom environment. It has already been shown that narrower size distributions and higher yields compared to batch synthesis can be achieved in synthesizing iron oxide nanoparticles using these reactors^24,25^. Despite the simplicity of the capillary reactors, they can be extended to multi-step reactions by direct injection of a further reagent into droplets, or droplet fusion *via* electrocoalescence^26^. This application has been shown using a three-step reaction for the synthesis of Au-Pd core-shell nanoparticles^27^.

A further advantage of microfluidic reactors is that they can be easily automated and real-time measurements of reaction properties easily integrated. Through this, microfluidic reactors that automatically screen, optimize, or monitor reaction conditions became a valuable tool, as they can be used as an inexpensive method for developing larger reactors^28^. An example of this can be seen of the microfluidic synthesis of lipid nanoparticles including an online fluorescent measurement controlling the particle quality using a feedback mechanism^29^. Continuous variation of reaction conditions in a microfluidic synthesis of gold nanoparticles was used to screen reaction conditions in a fast and automated manner^30^. Similar experiments were conducted for quantum dots in capillary reactors^31^. The online monitoring can also be combined with computer learning techniques to achieve a self-optimizing reactor^32^. It is demonstrated with a microfluidic reactor using an optimization algorithm to synthesize quantum dots of a pre-defined wavelength in a single step reaction^33^. Extending from our previous work on the synthesis of iron oxide nanoparticles in capillary reactors, here we show the synthesis of iron oxide/gold core-shell nanoparticles in a capillary, droplet reactor. Through the integration of an optical transmission measurement at the outlet of the reactor, an automated computer algorithm can be used to find the optimal synthesis conditions. The entire setup can be assembled for less than $100, allowing it to be used for initial screening experiments. To our knowledge, this is the first demonstration of an automated optimization of a multi-step core-shell nanoparticle synthesis in a capillary droplet reactor.

## Materials and Methods

### Fabrication of the multi-step droplet reactor

For the droplet capillary reactor, a 190 cm long piece of Tygon tubing (0.51 nm inner diameter, 1.52 nm outer diameter, Sigma-Aldrich, USA) was used into which several holes were punched using syringe needles. Into the first two holes, 15 cm from the beginning of the tubing, two fused silica capillaries (100 µm inner diameter, 360 µm outer diameter, Supelco, USA) were inserted that they formed a 90° angle in the center of the Tygon tubing. Furthermore, single capillaries were inserted at 100, 130, and 160 cm perpendicular to the center of the Tygon tubing for the addition of gold precursor. After insertion of the fused silica capillaries, the holes were sealed using a hot melt adhesive. At the outlet of the capillary droplet reactor, the optical transmission was measured using a light emitting diode (LED, 585 nm main emission wavelength), a photoconductor, and a pair of lenses to focus on the center of the tubing (Supplemental Materials S1, 2), all housed in a custom three-dimensional (3D) printed housing. A custom written Python script was used for transmission measurements, running of the optimization algorithm, and manipulation of the flowrate (Supplemental Material S3). For temperature control of the reactor, the entire assembly was submerged in a temperature controlled water bath at 70 °C.

### Synthesis of iron oxide/gold core-shell nanoparticles

For the synthesis of the core-shell nanoparticles, iron oxide nanoparticles were firstly synthesized within the droplets of the reactor, as previously described^24,34^. Briefly, through the first two fused silica capillaries of the reactor, two aqueous solutions were injected at a flow rate of 10 µL/min. The first solution was 0.06 M of FeCl_3_·6H_2_O (Alfa Aesar, USA) and 0.03 M of FeCl_2_∙4H_2_O (Sigma-Aldrich, USA) dissolved in DI water, and the second solution was 4 M ammonia (Alfa Aesar, USA). As a continuous phase mineral oil (M5904, Sigma-Aldrich USA) with 0.075 vol % Triton X-100 (Samchun Chemical, Korea) and 1.75 vol % Abil EM 90 (Evonik Industrial, Germany) was used, the flowrate injected through the central Tygon tubing was 10 µL/min. For the synthesis of the gold shell around the iron cores, a gold precursor solution was injected into the existing droplets through the single capillaries at 100, 130, and 160 cm. The gold precursor solution consisted of 0.03 M HAuCl_4_ (Sigma-Aldrich USA) dissolved in DI water. For all three injections, the same flowrate was used and flowrate was determined by a self-optimizing algorithm based on the transmission of the droplets and two initial guesses for the flowrate. To quench the reaction, core-shell nanoparticles were collected in a vial filled with water, once they left the capillary droplet reactor.

### Analysis of core-shell particles

After synthesis, the supernatant was removed once core-shell particles were collected from solutions using a permanent magnet. The resulting particles were washed with water and again separated out by magnet until the supernatant remained clear. The resulting particles were freeze-dried overnight and re-suspended in water at a concentration of 1 mg/mL. For transmission electron microscopy (TEM), the nanoparticle suspension was added to copper grids (Electron Microscopy Science, USA) and dried overnight. Afterwards, TEM images were taken using a JEM-2100F (JEOL, Japan) and particle sizes were analyzed using Image J (National Institute of Health, USA). Furthermore, energy-dispersive X-ray spectroscopy (EDS) was performed using the same equipment to determine the elemental composition of the nanoparticles. Absorption spectra of the nanoparticles suspension were taken using an UV-VIS spectrometer (Shimadzu, Japan), Zeta potentials were determined using a Zetasizer Nano ZS (Malvern Panalytical, United Kingdom), and MRI was taken at different particle concentrations using a 9.4T (400 MHz) MRI scanner (Agilent Technologies, USA). The photothermal effect of the core-shell nanoparticles in solution was tested by recording the temperature of solutions at different concentrations, while irradiating using a 630 nm light source (0.1 W/cm^2^, U-RFL-T, Olympus, Japan). Lastly, SERS effect was measured by incubating the iron oxide/gold core-shell nanoparticles overnight in a 10^−4^ M solution of 4-Aminothiophenol (4-ATP, Sigma-Aldrich, USA), and separating the particles afterwards using a magnet. Raman spectra of the 4-ATP coated particles and 4-ATP powder were taken using Raman spectra (LabRam Aramis, Horriba Jovin Yvon, Japan).

## Results and Discussion

### Multi-step capillary droplet reactor

**Figure 1:**
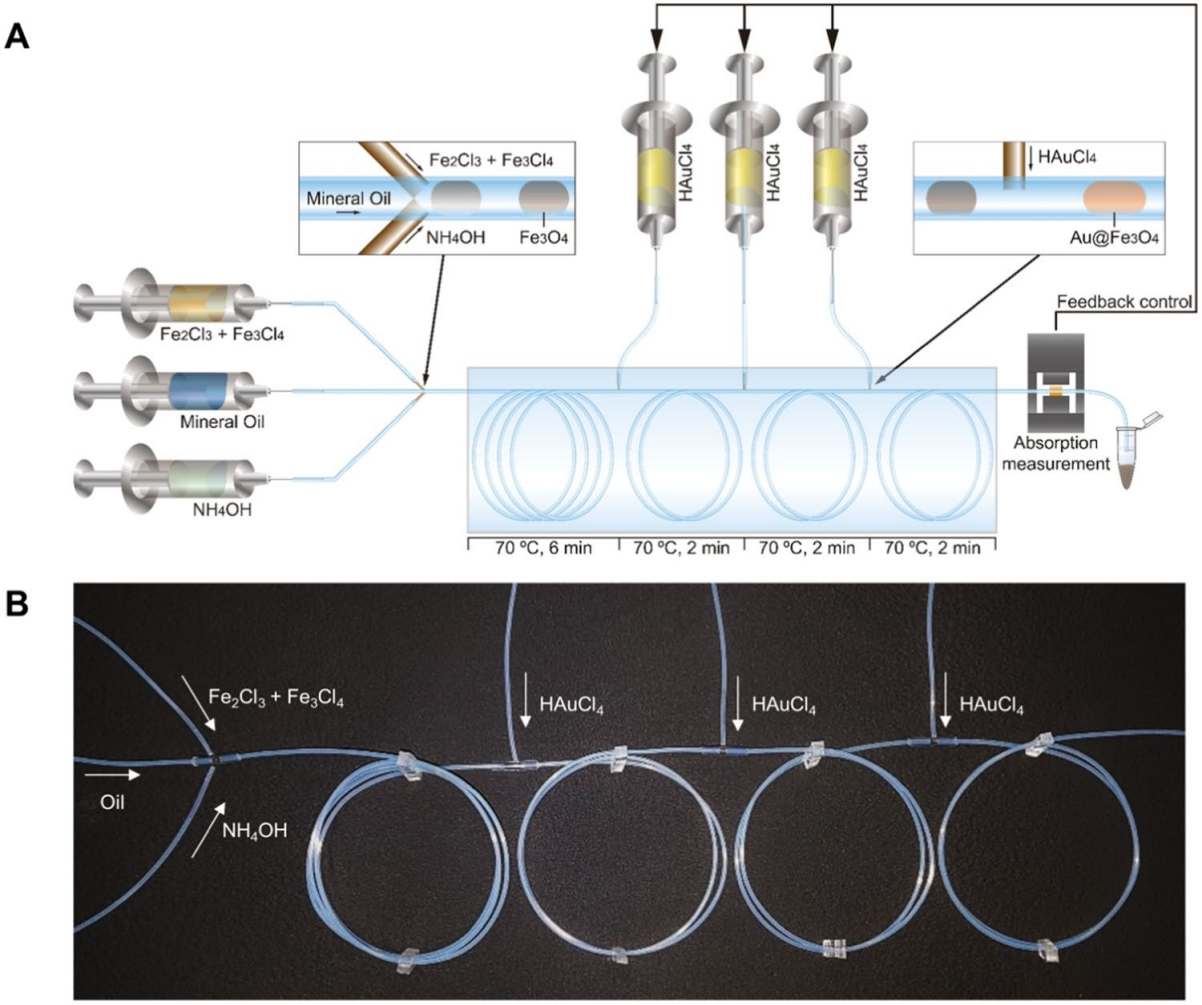
Schematic of the multi-step, capillary droplet reactor consisting of the central Tygon tubing, the first capillary junction for the generation of droplets, the three consecutive junctions for the injection of gold precursor solution into the droplets, and the transmission measurement at the outlet of the reactor (**A**). Photograph of the Tygon tubing used for the reactor showing the first junction for the generation of droplets and the following three reagent injection junctions (**B**).

**Figure 2:**
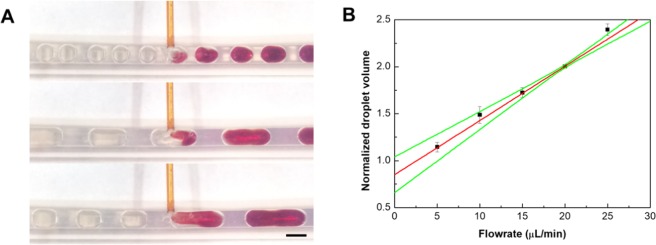
Photographs of injecting a reagent into droplets using a capillary junction. For illustration purpose, droplets contain a potassium cyanide solution to which an iron(III) chloride solution is added. Upon mixing, the two reagents form a red complex, indicating fast mixing within the droplets. The scale bar is 1 mm (**A**). Graph of droplet volume after injection of the additional reagent normalized to the original droplet volume against the flowrate of the additional reagent (**B**).

A capillary droplet reactor was used for the feedback controlled synthesis of iron oxide/gold core-shell nanoparticles (Fig. 1). The reactor consisted of a Tygon tube in which droplets were generated from an iron precursor solution and a base for the synthesis of iron oxide nanoparticles (Supplemental Video S1). After an initial incubation of the droplets for the formation of iron oxide nanoparticles, a gold precursor solution was injected into the droplets in three separate steps, each followed by an incubation period. Through the iterative addition of gold precursor, gold nucleation sites can be formed on the iron oxide particles first^7^, and subsequently grown into a gold shell^35^. As the droplet generation performance was already characterized in our previous paper for the synthesis of iron oxide nanoparticles^24^, only the merging performance of the capillary device was tested before synthesis experiments (Fig. 2). It was possible to inject a further reagent into the droplet through an additional capillary. The internal convention inside of the droplets lead to a fast mixing inside of the droplets and thus a homogenous reagent concentration was achieved (Fig. 2A, Supplemental Video S2). A linear relation between the droplet volume after injection and the flowrate of the additional reagent could be observed to a flowrate of 30 µL/min (Fig. 2B). Above this flowrate, secondary droplets were generated in between the original droplets, just containing the secondary reagent injected by the capillary. Therefore, we finally optimized a maximum flowrate of 30 μL/min for all injections through additional capillaries.

### Transmission measurement and self-optimizing algorithm

**Figure 3:**
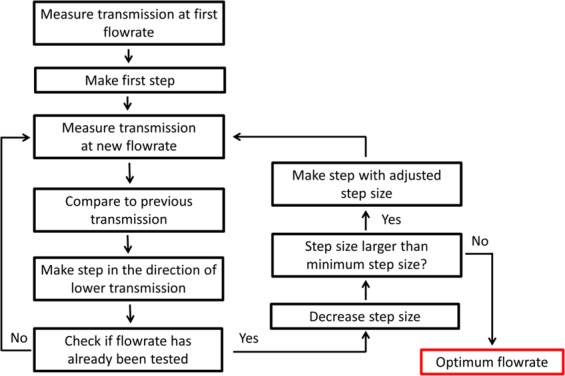
Schematic of the simplex optimization algorithm used for finding the optimum synthesis conditions.

**Figure 4:**
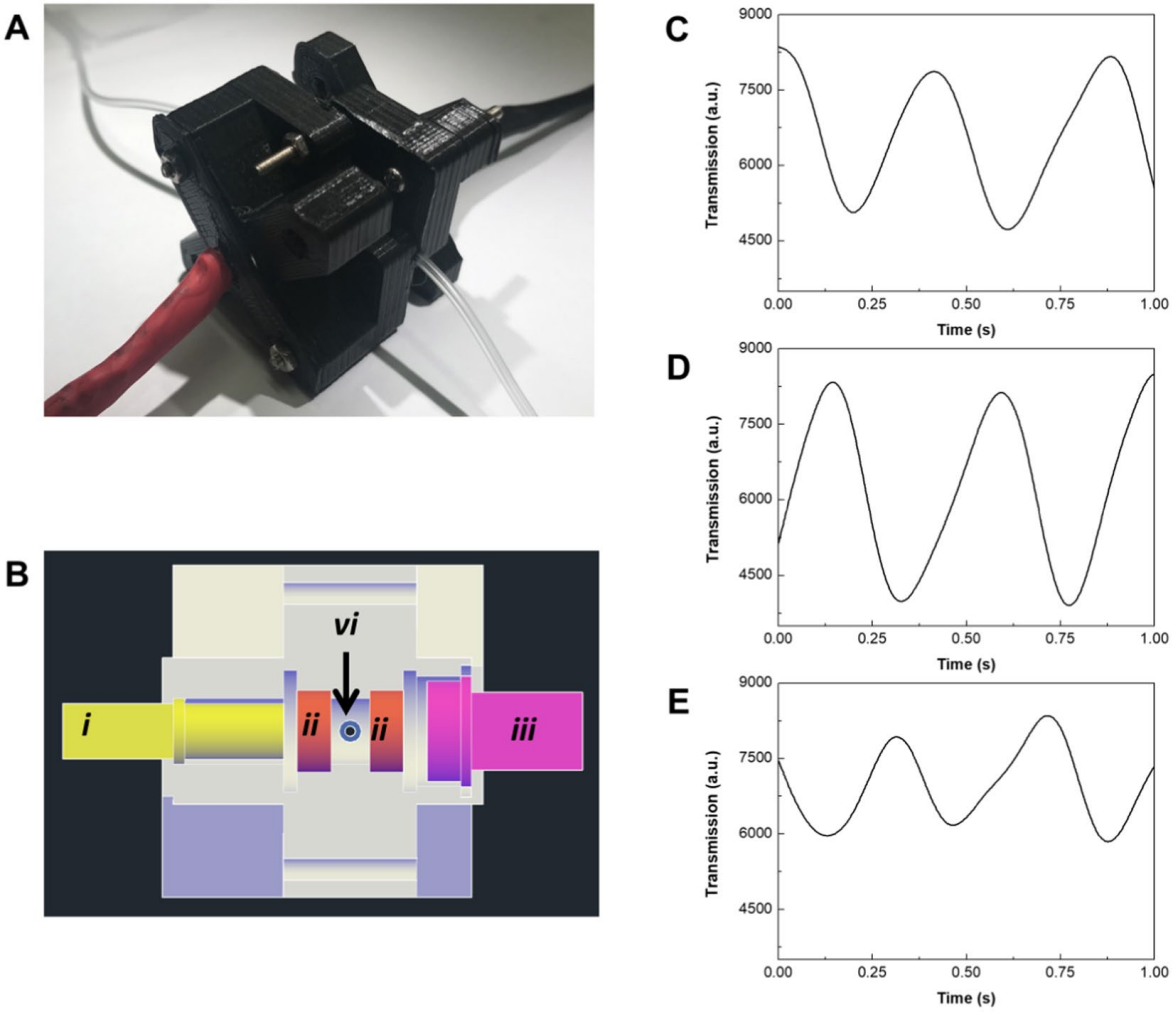
Photograph of the assembled transmission measurement device (**A**). Cross-section of the transmission measurement device with the LED (i), two lenses (ii) to focus on the droplets in the Tygon tubing (iv), and the photoconductor (iii) for detection (**B**). Examples of collected droplet transmission data for a flowrate of the gold precursor of 5 (**C**), 10 (**D**), and 20 µL/min (**E**).

**Figure 5:**
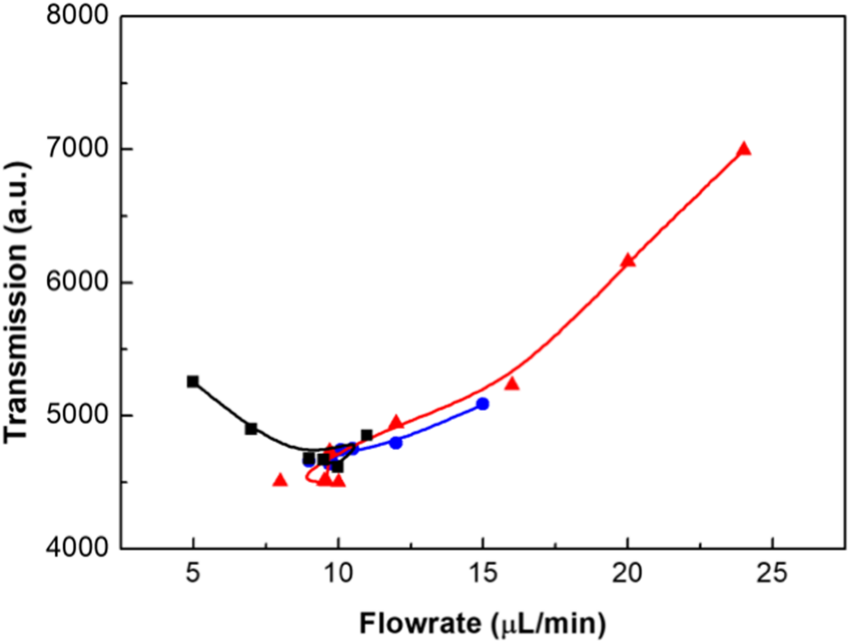
Graph of three separate experiments with the self-optimizing algorithm starting from different initial guesses and initial step sizes. The initial guesses are 5, 15, and 24 µL/min with step sizes of 2, 3, and 4 µL/min for the black, blue, and red curve, respectively.

A computer algorithm was used in combination with the capillary droplet reactor and a transmission measurement to enable the reactor to find optimum synthesis conditions for the iron oxide/gold core-shell nanoparticles in an automatic manner. Based on the Simplex algorithm^36^, our algorithm started from two initial guesses and changed the flowrate of gold precursor solution until the transmission reached a minimum (Fig. 3). Once this minimum has been passed, the step size was decreased by the algorithm to precisely determine the optimum conditions. In experiments, a minimum step size of 0.1 μL/min was used. However, due to instabilities of the flowrate originating from the syringe pumps and noise of the transmission measurement, the accuracy of the experimental setup is approximately ±0.2 µL/min. The maximum step size is determined by the two initial guesses used. For the purposes of a microfluidic droplet reactor, it is beneficial as large pressure changes due to changing flowrates can be prevented, ensuring stable conditions for droplet generation. To measure the optical transmission of the droplets containing the core-shell nanoparticles, a custom 3D printed assembly containing a LED and a photoconductor was used (Fig. 4A,B). Furthermore, two lenses were included in the assembly to focus the light on the center of the tubing containing the droplets. A LED with a central wavelength of 585 nm was used to match the expected absorption characteristics of the iron oxide/gold core-shell nanoparticles. Depending on the flowrate of the gold precursor, the thickness of the gold shell varied leading to different absorption of the core-shell nanoparticles (Fig. 4C–E). In addition, a high flowrate of the gold precursor solution lead to the formation of gold nanoparticles as a side product, which had different absorption characteristics compared to the core-shell nanoparticles. Transmission data collected over a time span of 30 seconds is shown in the Supplemental Material S4. For the optimization algorithm droplet transmission data was collected for two minutes and the droplet data extracted using a threshold, the averaged droplet data was then used for the Simplex algorithm. Experiments were conducted with the self-optimizing capillary droplet reactor and different starting conditions (Fig. 5). From all three initial guesses, the algorithm converged to an optimal flowrate of 9.7 ± 0.2 µL/min in less than 10 iterations of the algorithm (Supplemental Material S5). This was done without requiring any manual inputs into the system in a time of 30 minutes or less for each starting condition.

### Characterization of iron oxide/gold core shell nanoparticles

**Figure 6:**
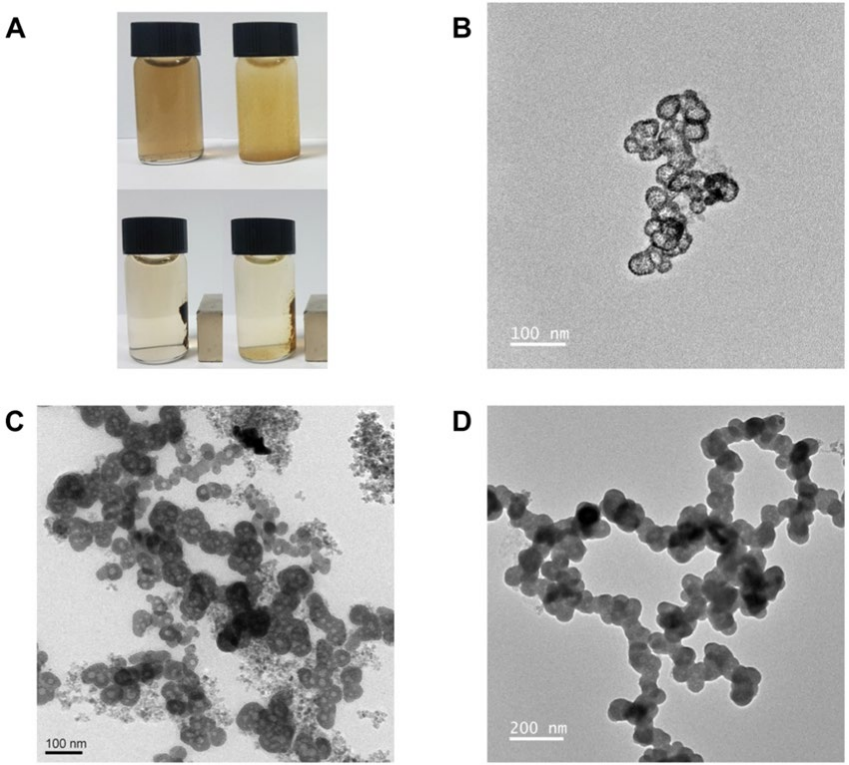
Photograph of iron oxide nanoparticles and iron oxide/gold core-shell nanoparticles in solution (top, **A**) and after separation with a magnet (bottom, **A**). TEM images of iron oxide/gold core-shell nanoparticles synthesized at different flowrates of gold precursor. Starting from initial growth of gold nuclei on the surface of the iron oxide core particles (5 μL/min, **B**), over a closed shell (10 μL/min, **C**), to a thick gold shell (20 μL/min, **D**).

**Figure 7:**
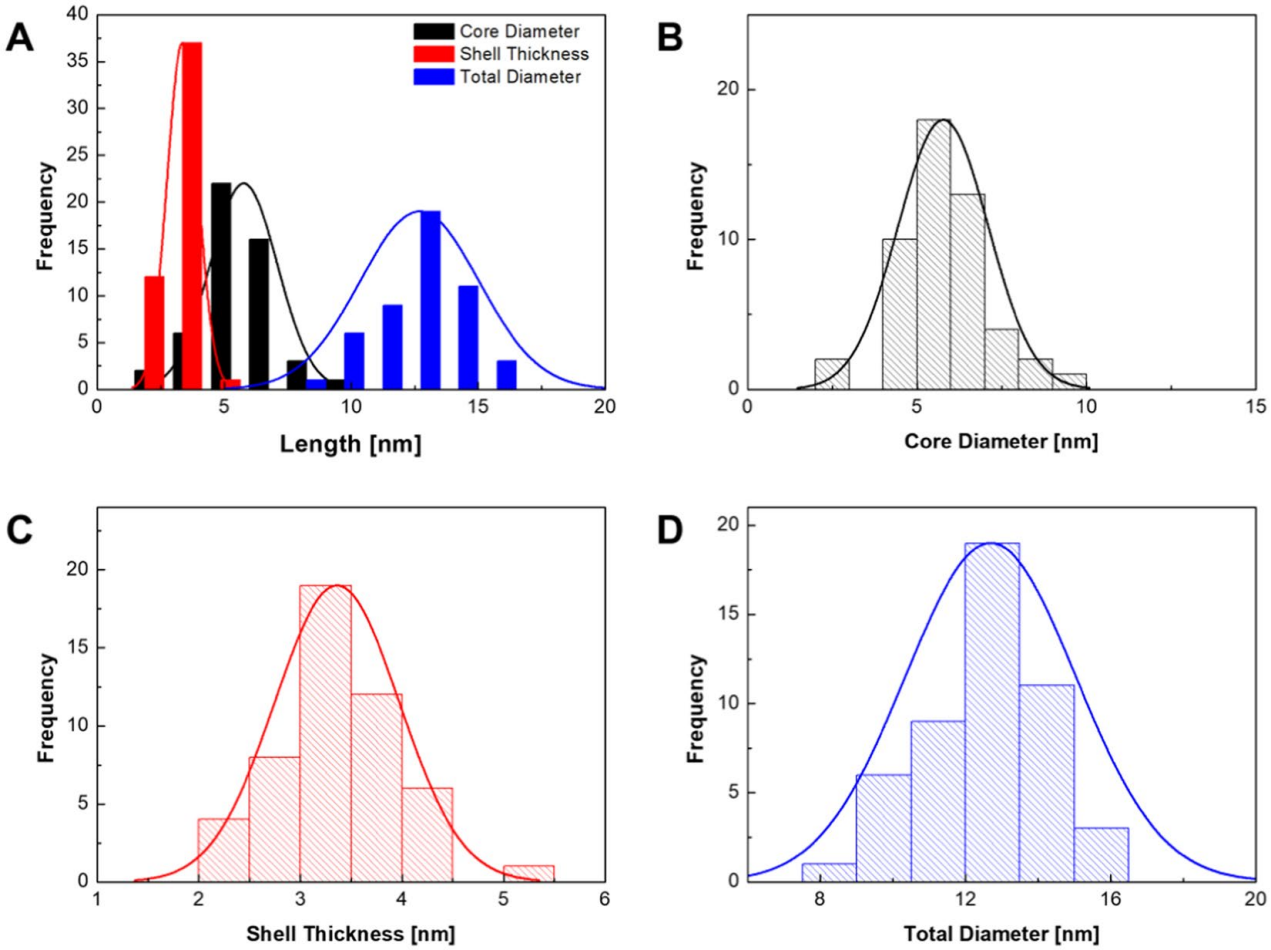
Histograms of the diameter of iron oxide core particles, thickness of the gold shell, and the total diameter (**A**). Individual histograms of iron oxide core diameter (**B**), gold shell thickness (**C**), and total diameter of the core-shell nanoparticles (**D**).

**Figure 8:**
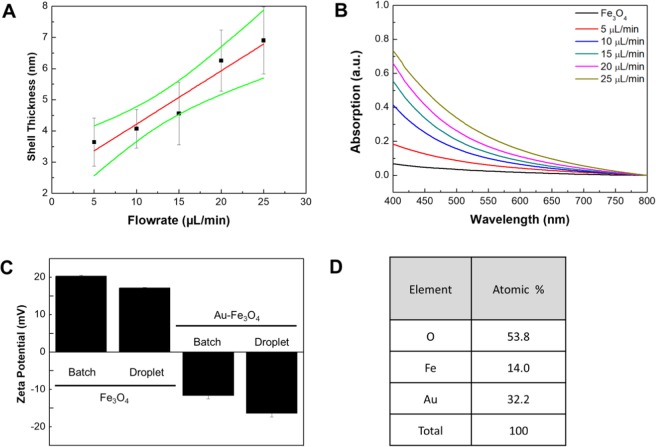
Graph of gold shell thickness against flowrate of the gold precursor during synthesis (**A**). UV-VIS spectra of core-shell particles for different flowrates of gold precursor (**B**). Measured Zeta potentials for iron oxide nanoparticles and iron oxide/gold core-shell nanoparticles synthesized in the droplet capillary reactor and comparative batch reactions (**C**). Elemental composition of core-shell nanoparticles according to EDS spectroscopy (**D**).

The synthesis conditions of the iron oxide core particles were chosen as previously described^24^. Through the addition of a gold shell around the particles, the color of the particles in solution changed from brown to yellow, and later to a dark red for thicker gold shells (Fig. 6A, Supplemental Material S6A). While at low flowrates of the gold precursor solution the supernatant remained clear after separation with a magnet, the supernatant at higher flowrates of gold precursor remained turbid, indicating the formation of gold particles in a side reaction. In TEM images, the formation of the gold shell could be observed. At low flowrates of the gold precursor, the formation of several gold nuclei on the surface of the iron oxide nanoparticles could be observed (Fig. 6B). With increasing flowrate of the gold precursor, the nuclei kept growing until they eventually formed a closed shell around the iron oxide core particles (Fig. 6C). At this stage, further addition of the gold precursor lead to the gold shell growing thicker (Fig. 6D). From the TEM images, the distributions of particle size were determined at the optimum flowrate of 9.7 μL/min (Fig. 7). At this condition, the diameter of the iron oxide core was 5.8 ± 1.4 nm, the thickness of the gold shell was 3.5 ± 0.6 nm, and the total diameter of core-shell particles 13.1 ± 2.5 nm. The elemental analysis of the core-shell nanoparticles by EDS revealed a composition of ~10% by mass for iron and oxygen and the remaining 80% gold (Fig. 8D). Furthermore, Zeta potentials were measured of the iron oxide core particles, and the iron oxide/gold core shell nanoparticles in batch and droplet based synthesis (Fig. 8C). Before the addition of the gold shell, the iron oxide core particles showed a positive Zeta potential, however, through the addition of the gold shell the core-shell particles gained a negative surface charge, further indicating the successful synthesis of the gold shell around the iron oxide core nanoparticles. Lastly, the effect of gold precursor flowrate on the thickness of the gold shell and the absorption spectra of the particles was determined (Fig. 8A,B). As can be expected, a linear relationship between the flowrate of the gold precursor and the thickness of the gold shell was observed. With increasing thickness of the gold shell, the absorption of the particles in the wavelength range from 400 to 700 nm was also increased, providing further evidence for the increased thickness of the gold shell.

### Applications of iron oxide/gold core-shell nanoparticles

**Figure 9:**
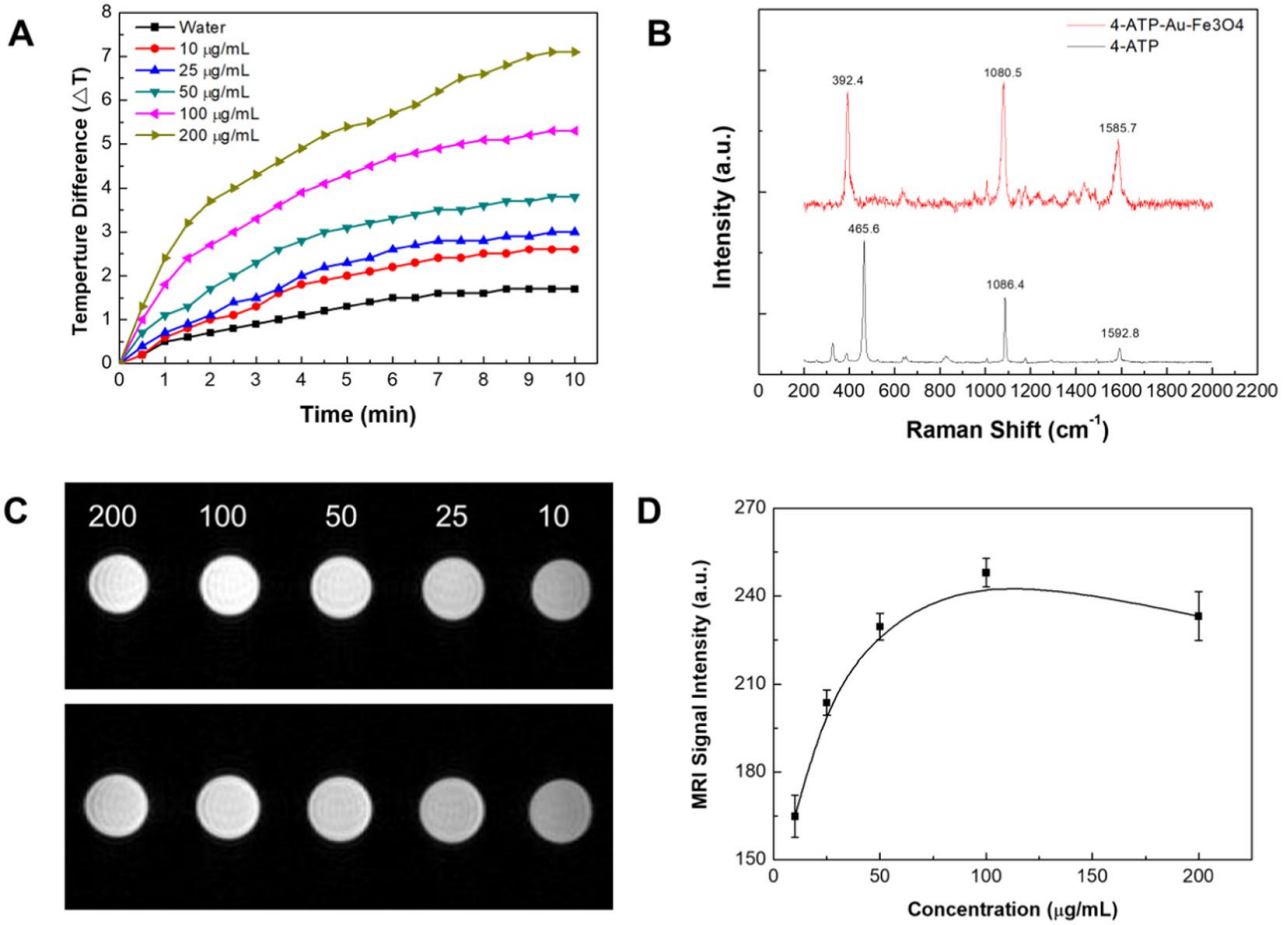
Temperature as a function of time when irradiating different concentrations of core-shell nanoparticle solutions with light demonstrating photothermal therapy applications (**A**). Raman spectra of 4-ATP and 4-ATP on the surface of iron oxide/gold core-shell nanoparticles, illustrating SERS effect (**B**). MRI images of different concentrations of core-shell nanoparticle solutions taken at two different heights (**C**) and graph of extracted MRI signal strength as a function of nanoparticle concentrations (**D**).

Three different applications and their versatility were demonstrated using the iron oxide/gold core-shell nanoparticles. First, the photothermal effect of the gold shell was demonstrated (Fig. 9A). During illumination with a light source of matching emission wavelength, the temperature of solutions containing the core-shell nanoparticles significantly increased as compared to a water control sample. At higher concentrations of 100 and 200 μg/mL, temperature increases of 5 and 7 °C can be achieved after 10 minutes. These temperature increases would be sufficient for the core-shell nanoparticles to be used in photothermal therapy. By replacing the light source (0.1 W/cm^2^) with a more powerful one, it would be possible to achieve similar temperature increases with lower concentrations. Second, the application of the iron oxide/gold core-shell nanoparticles to SERS was demonstrated (Fig. 9B). In the Raman spectrum, distinct peaks can be observed with the peak at 1592 cm^−1^ corresponding to C–C stretching vibrations, the peak at 1086 cm^−1^ corresponding to C–S stretching vibrations, and the peak at 465 cm^−1^ corresponding to C–C–C bending vibrations^37,38^. Due to the absorption of the 4-ATP onto the gold surface, the shifts in wavelength can be observed to show SERS activity of the iron oxide/gold core-shell nanoparticles^39^. Third, an enhancement of the peak intensity can be seen, most clearly visible with the peak at 1585 cm^−1^, but also through the peaks at 1473 cm^−1^ (C–C stretching), 1175 cm^−1^, (C–H bending), and 1004 cm^−1^ (C–C bending)^40^. In addition, the iron oxide/gold core shell nanoparticle was used as a contrast agent for MRI. For this purpose, MRI of solutions containing different concentrations of core-shell nanoparticles was taken and the increase in signal strength was measured (Fig. 9C,D). Up to a concentration of 100 μg/mL, the MRI signal was increased, plateauing for higher concentrations before eventually decreasing (Supplemental Material S6B,C). The decrease in MRI signals at high concentrations of nanoparticles can be explained by the core-shell nanoparticles, forming agglomerates. As the position of the plateau marking the strongest MRI signal coincides with the concertation ideally used for photothermal therapy, the material is suitable for theranostics as the same dose of nanomaterial can be used for therapy and monitoring. It illustrates how particles synthesized using the presented droplet reactor can be used for a number of different applications with possible theranostic applications combining MRI contrast agent and photothermal therapy in a single core-shell nanoparticle.

## Conclusions

Here, we have shown the design and testing of a self-optimizing, capillary droplet reactor for synthesizing core-shell nanoparticles in a multi-step reaction. With this reactor, iron oxide/gold core shell particles were successfully synthesized. The optimization algorithm, starting from three different guesses, arrived at the same conditions in less than 10 iterations each time, taking less than 30 minutes for each experiment. Furthermore, the application of the iron oxide/gold core-shell nanoparticles was demonstrated for SERS, and as a theranostic material for combining photothermal therapy and MRI contrast agent in a single material. Therefore, the capillary droplet reactor could be particularly useful in the automatic screening of synthesis conditions or automatized optimization of reaction conditions on a laboratory scale.

## Supplementary information

Supplementary Information.
