# Supplementary material for: Automated droplet reactor for the synthesis of iron oxide/gold core-shell nanoparticles
Source: Sci Rep. 2020 Feb 3;10:1737. doi: 10.1038/s41598-020-58580-9 (PMC6997455; doi:10.1038/s41598-020-58580-9)
Supplement: Supplementary file 1 — Supplementary Information. [file 41598_2020_58580_MOESM1_ESM.docx]

**Supporting Information**

**Automated droplet reactor for the synthesis of**

**iron oxide/gold core-shell nanoparticles**

Christian D. Ahrberg^‡^, Ji Wook Choi^‡^ and Bong Geun Chung^*^

Department of Mechanical Engineering, Sogang University, Seoul, Korea

**
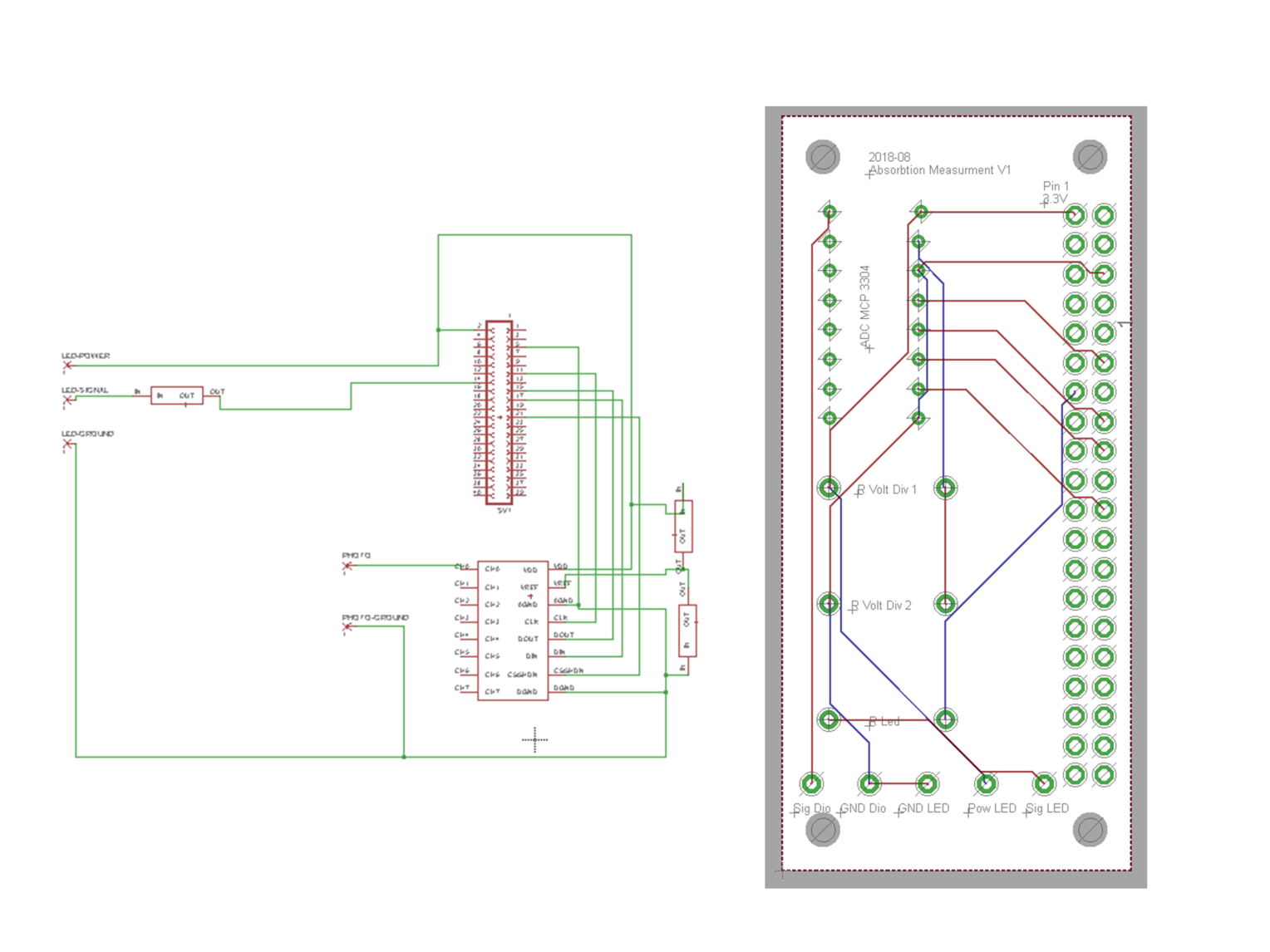
**

**Supplemental Material S1.** Schematic of the transmission measurement device (left) and drawing of the printed circuit board layout (right).


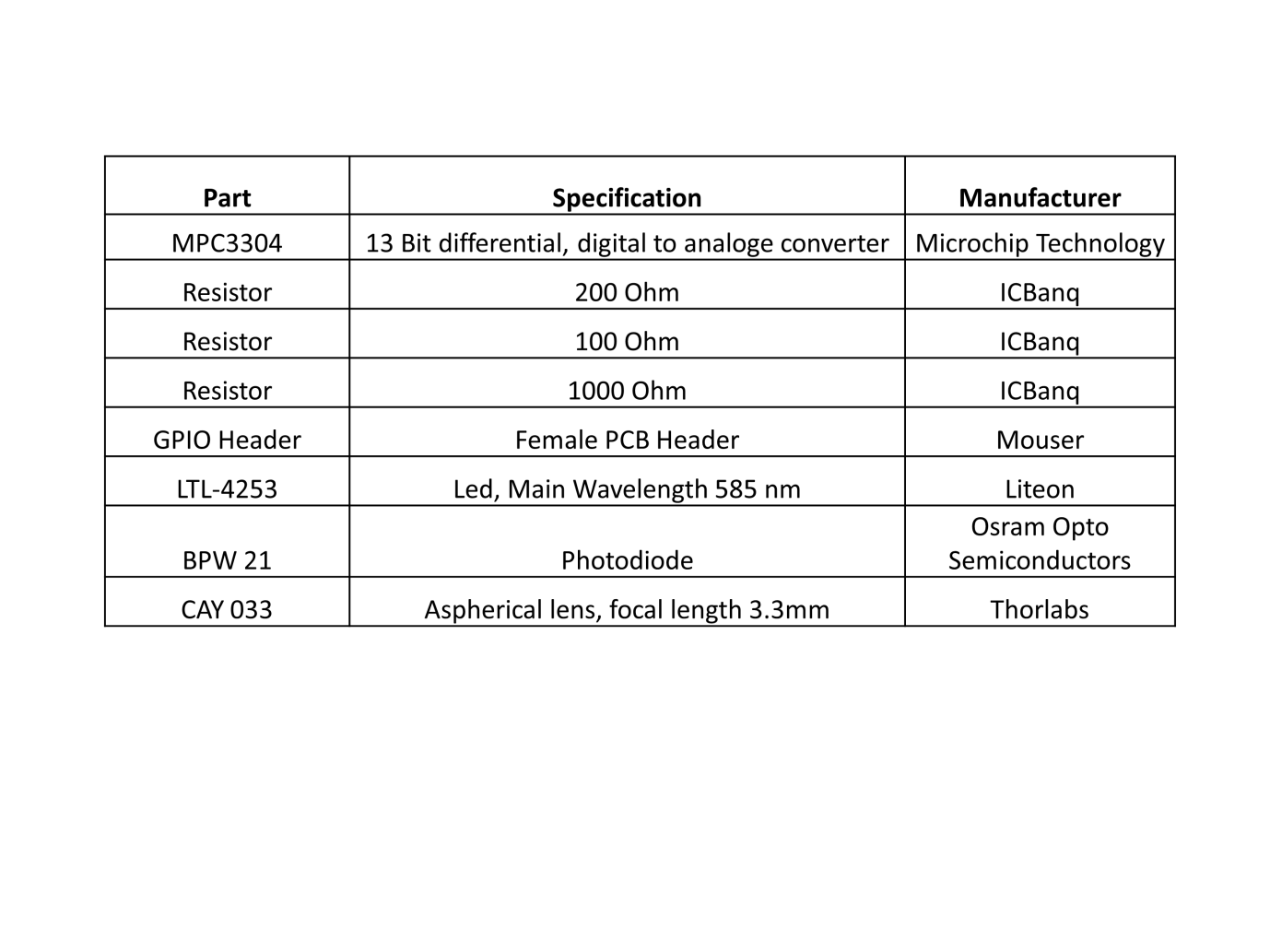


**Supplemental Material S2.** Table of electronic components used for the transmission measurement, including specifications and suppliers.

**Supplemental Material S3.** Python code for transmission measurement and manipulation of flowrates. The custom written Python code can be found in the Github under the following link: <https://github.com/cDNAg1/Core-Shell-nanoparticles>. The code is written to be run using Python version 2.7 (for version 3 the commands send to the syringe pumps have to be modified) using a Raspberry pi single board computer. The code starts the syringe pumps and waits for a given time for the conditions to stabilize (during this time transmission data is also collected, but not used for analysis). Afterwards, the algorithm collects transmission data, analyzes it, and determines the next flowrate as outlined by the Simplex algorithm. All transmission data is saved to text files, for later manual analysis if so desired.


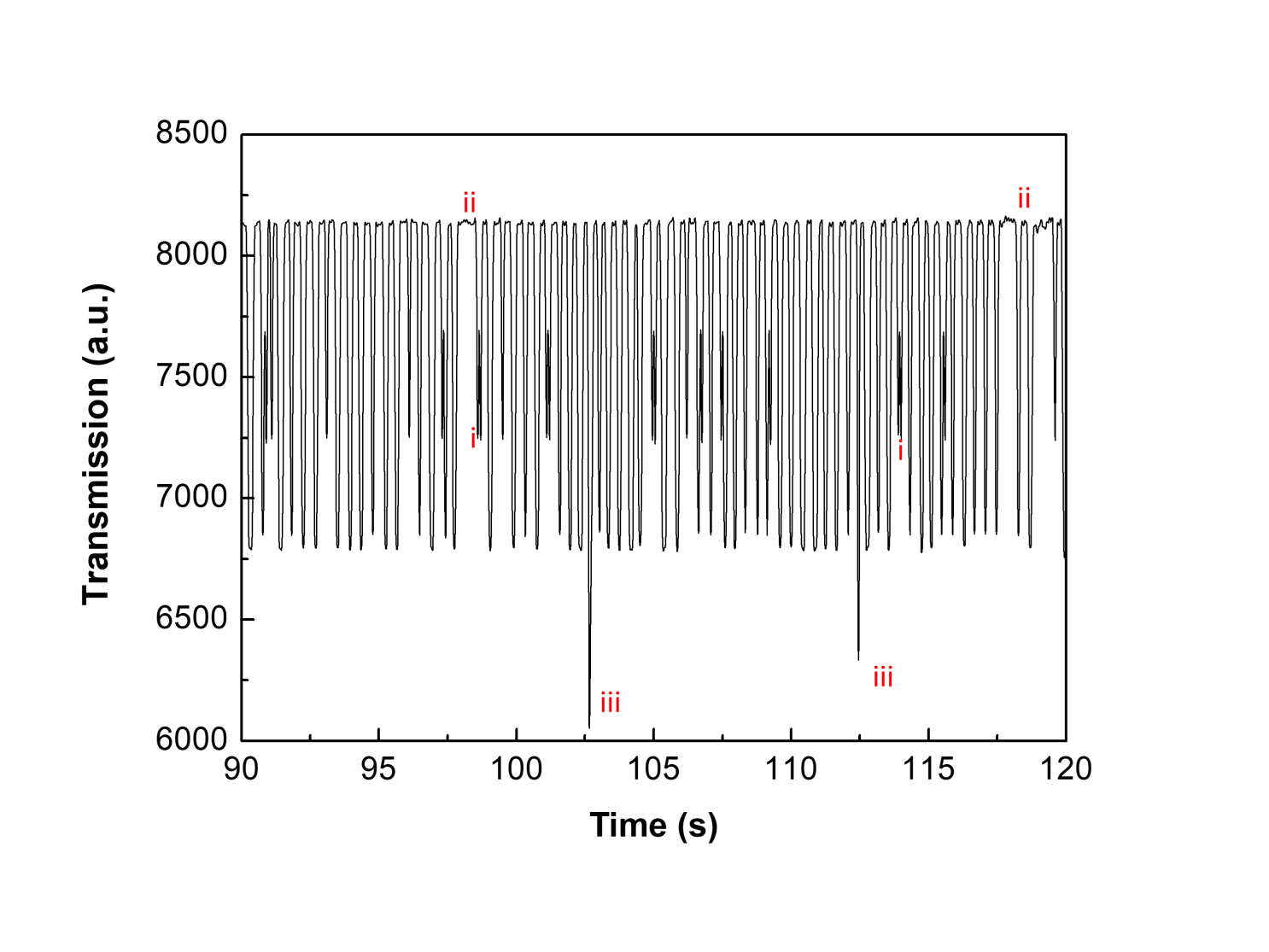


**Supplemental Material S4.** Graph showing 30 seconds of transmission data displaying droplet merging (i), absence of droplets (ii), and random noise from detector (iii).


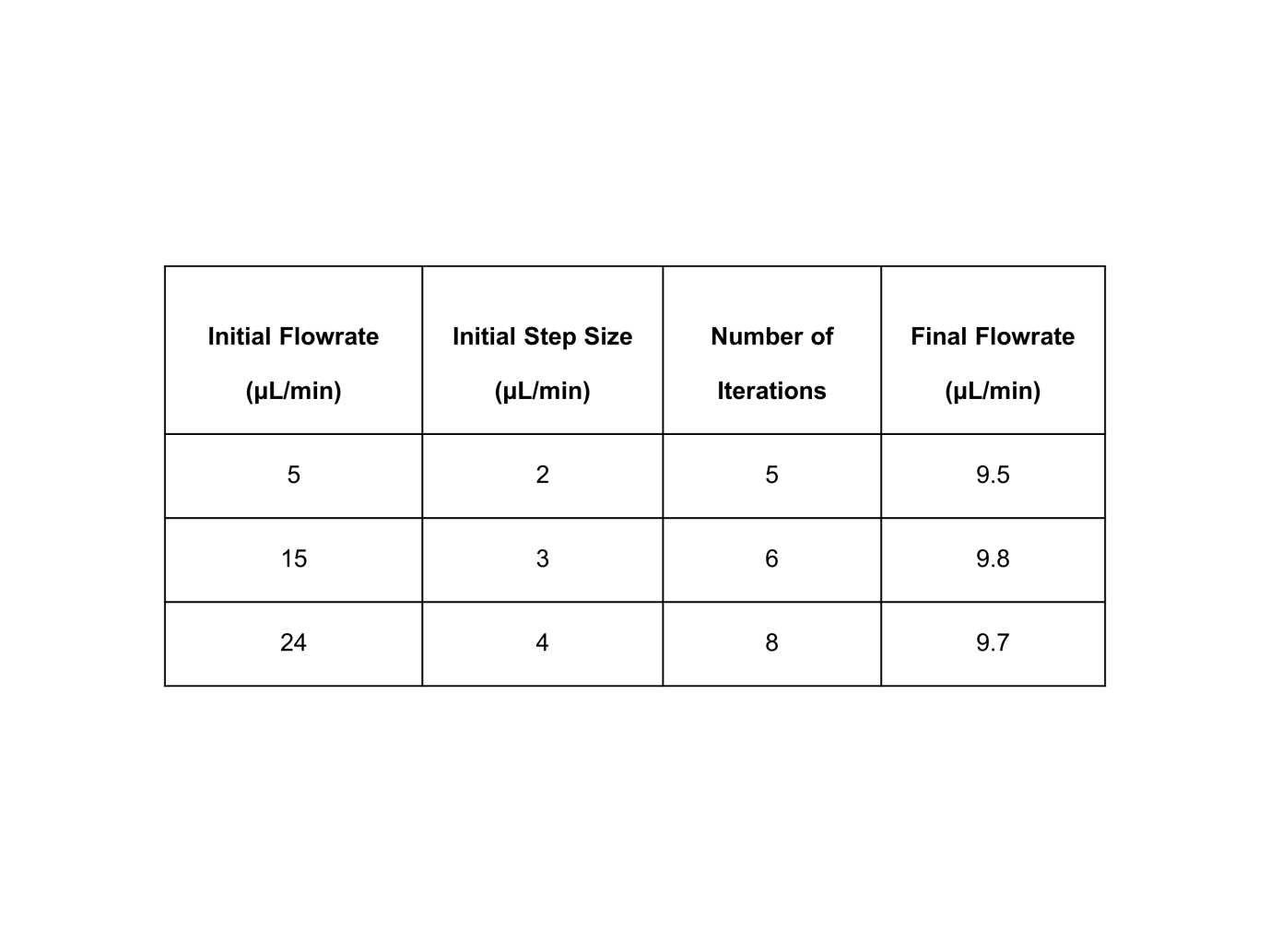


**Supplemental Material S5.** Table showing convergence data for the three self-optimization experiments conducted.


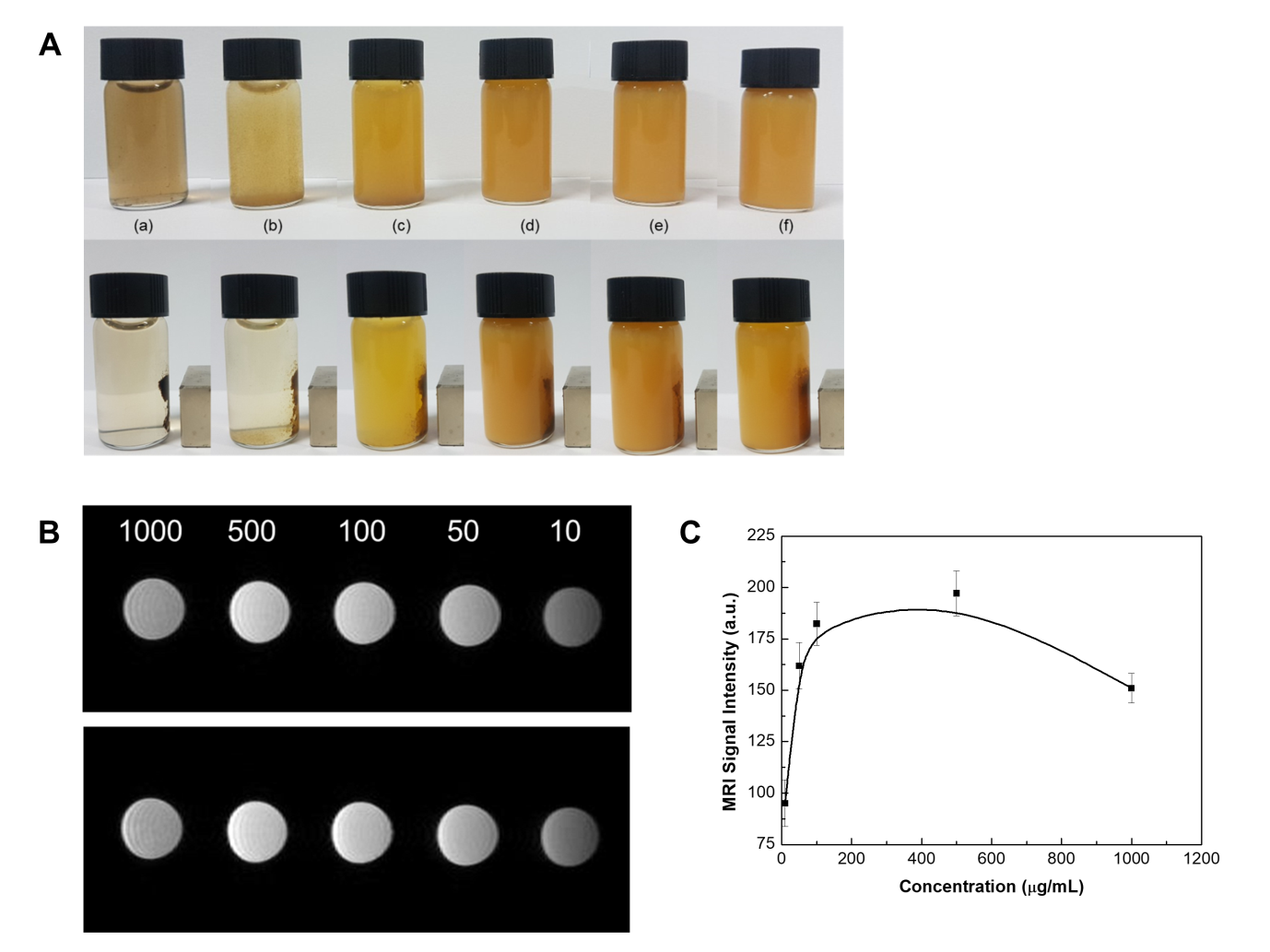


**Supplemental Material S6.** Photograph showing nanoparticles synthesized at a gold precursor flowrate of 0 (a), 5 (b), 10 (c), 15 (d), 20 (e), and 25 μL/min (f) in solution (top row, A), and after separation by magnet (bottom row, A). Two slices of MRI for different concentrations of iron oxide/gold core-shell nanoparticles (B). Graph of MRI signal strength for different concentrations of core-shell nanoparticles (C).

**
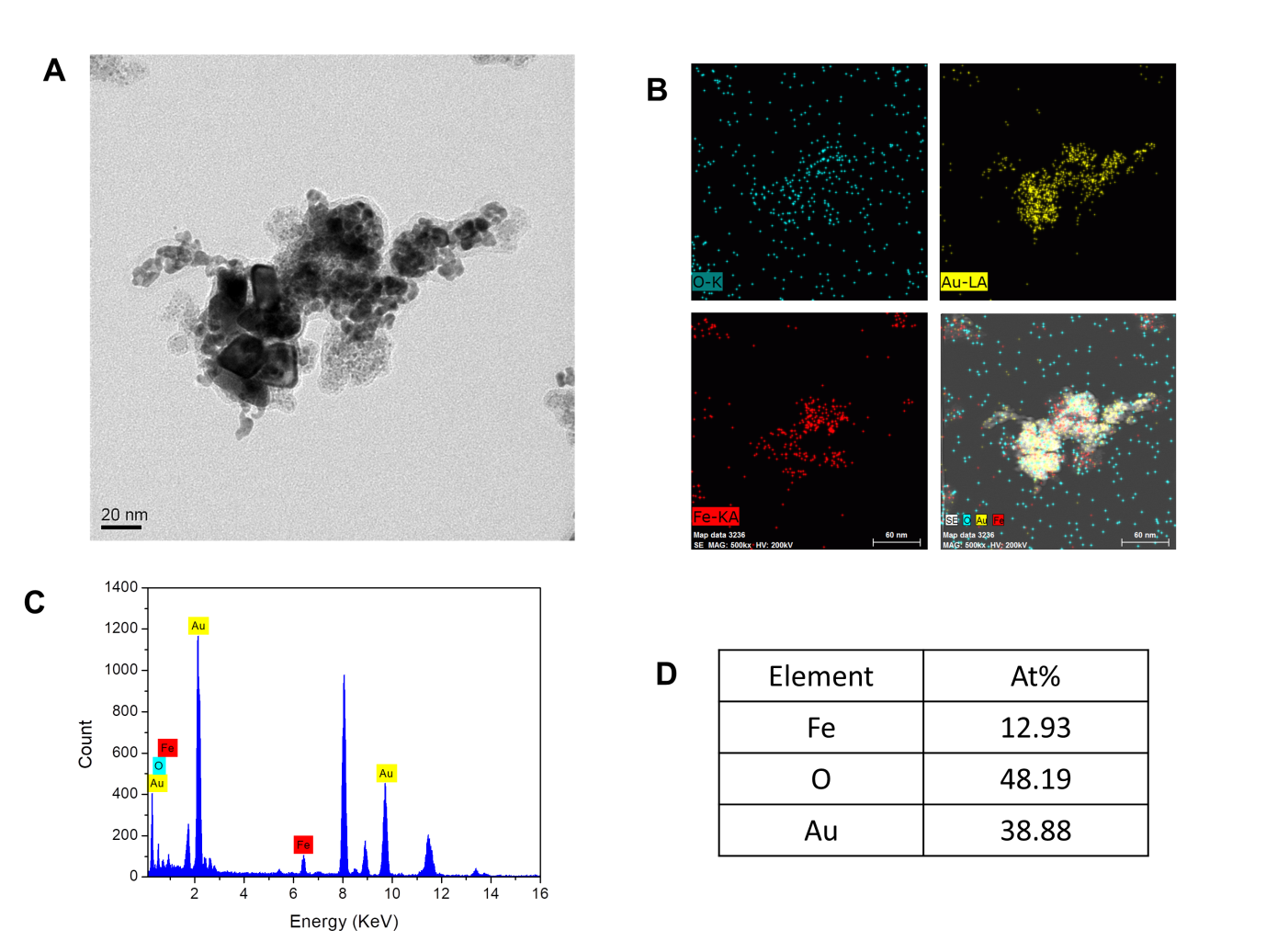
**

**Supplemental Material S7.** TEM of synthesized core-shell nanoparticles (A), elemental mapping of oxygen, gold, and iron as well as overlay of the three images showing overlapping of the iron and the gold signals (B). XRF spectrum of tested nanoparticles (C), and extracted elemental composition of the tested core-shell nanoparticles (D).

**Supplemental Video S1.** Video of droplet generation at the initial junction of two fused silica capillaries in the Tygon tubing. For this video, the continuous phase consists of mineral oil with 0.075 vol % Triton X-100 (Samchun Chemical, Korea) and 1.75 vol % Abil EM 90 (Evonik Industrial, Germany). For the dispersed phase, water was used for both inlets. The video is recorded in a real-time manner.

**Supplemental Video S2.** Video of droplet merging at the first droplet merging junction in the Tygon tubing. For illustration purpose, the initial droplets are filled with a solution of KSCN, and a FeCl_3_ solution is injected into them. Upon injection of the second reagent into the droplets, a red iron complex is formed visible in the video, illustrating the mixing characteristics. The video is recorded in a real-time manner.
